# Additive effectiveness of autologous platelet-rich fibrin in the treatment of intrabony defects: A PRISMA-compliant meta-analysis

**DOI:** 10.1097/MD.0000000000014759

**Published:** 2019-03-15

**Authors:** Ang Li, Hongjie Yang, Jingyi Zhang, Shulian Chen, Hongqiang Wang, Yanzheng Gao

**Affiliations:** a Henan Provincial People's Hospital, People's Hospital of Zhengzhou University, School of Clinical Medicine, Henan University, Zhengzhou; b Department of Orthopedics, China Flat Coal Shenma Medical Group General Hospital, Pingdingshan, China.

**Keywords:** intrabony defects, meta-analysis, open flap debridement, platelet-rich fibrin

## Abstract

**Background:**

This meta-analysis was performed to determine the additive effectiveness of autologous platelet-rich fibrin in the treatment of intrabony defects in chronic periodontitis patients.

**Methods:**

Pertinent studies were identified by a search in Medline, EMBASE, the Web of Science, and the Cochrane Library. The trials searched were evaluated for eligibility. Cochrane Collaboration's Review Manager software was used to perform the meta-analyses.

**Results:**

Twelve eligible clinical trials were included. Pooled data found that adjunctive platelet-rich fibrin exactly yielded a significantly superior probing depth reduction compared with open flap debridement alone (weighted mean difference, 1.01; 95% confidence interval 0.95–1.08; *P* < .00001). The clinical attachment level (CAL) gain after treatment for 9 months was higher in patients treated with platelet-rich fibrin plus open flap debridement group than in open flap debridement-treated patients (weighted mean difference, 1.29; 95% confidence interval 0.96– 1.61; *P* < .00001). Similarly, the meta-analysis demonstrated that platelet-rich fibrin was superior to single open flap debridement with respect to gingival marginal level change (weighted mean difference, 0.45; 95% confidence interval 0.31–0.58; *P* < .00001). Regarding the hard tissue radiographic parameters, including defect depth reduction and percentage of fill defects in bone, adjunctive platelet-rich fibrin yielded significantly superior results compared with open flap debridement alone.

**Conclusion:**

Adjunctive use of platelet-rich fibrin with open flap debridement significantly improves fill defects when compared to open flap debridement alone. However, additional powered studies with much larger sample sizes are needed to obtain a more concrete conclusion.

## 1 Introduction

Periodontitis is an immunoinflammatory disease that is characterized by the destruction of the attachment apparatus of the periodontium.^[1]^ Untreated periodontitis results in progressive attachment loss that may eventually lead to early tooth loss. Periodontal tissue regeneration has always been a challenge for periodontists owing to its structural complexity. Currently, no single regenerative material is considered the gold standard for treatment. Various nonsurgical and surgical therapies form the basis of the periodontal treatment of furcation defects.^[1–3]^ The current concept of periodontal therapeutic goals includes not only the resolution of inflammation and infection but also the regeneration of lost anatomical structures to restore health and function. Otherwise, conventional open flap debridement (OFD) falls short of regenerating tissues destroyed by the disease.

Platelet-rich fibrin (PRF), a new generation of platelet concentrate developed by Choukroun in 2005, is a close congregation of platelets, circulating stem cells, cytokines, structural glycoproteins (such as thrombospondin-1), and glycan chains entangled in a complex fibrin meshwork that can be used as fibrin membrane.^[1,4]^ In recent years, the beneficial effects of PRF have been studied in various procedures, such as facial plastic surgery, in a sinus-lift procedure as a sole osteoconductive filling material and in multiple gingival recession cases treated with a coronally displaced flap.^[5]^ Owing to containing all the constituents of blood favorable for healing and immunity, it has been proposed that PRF is a healing biomaterial that accelerates wound closure and mucosal healing, along with a significant diminution of pain and discomfort. Many studies found that PRF is efficacious clinically and radiologically in the treatment of intrabony defects after the enucleation of various periapical lesions in the treatment of chronic periodontitis patients,^[2,6–8]^ but further critical evidence is needed to complete clinical application guidance.

Hence, the aim of this updated meta-analysis was to systematically evaluate the additive effectiveness of autologous PRF in the treatment of intrabony defects of chronic periodontitis patients when used along with OFD in terms of clinical and radiological outcomes.

### 1.1 Inclusion and exclusion criteria

PRISMA guidelines were followed for the inclusion of studies^[9]^ in this systematic review and associated meta-analyses, and a detailed description of the inclusion criteria are as follows:

1. trials had to be properly randomized;
2. no additional agents or interventions confounded the comparison;
3. contain patients with histologically proven intrabony defects of chronic periodontitis;
4. patients included in the trials should have no systemic diseases that could potentially influence the outcome of periodontal therapy.

The exclusion criteria were as follows:

1. the study only featured comparisons of other types of chemotherapy regimens;
2. early studies published as a series of articles from the same institution or author that contained significant overlapping data were excluded for fear of multiple publication bias;
3. case reports, editorials, experimental studies, conference articles, and other studies that failed to provide detailed results were excluded.

### 1.2 Literature search

Both published and unpublished studies were searched for publication bias. The following electronic databases were extensively searched from their inception through November 2017 independently by 2 investigators: EMBASE, Medline, the Cochrane Library, the Web of Science with keywords centered on the terms “platelet-rich fibrin”, “open flap debridement”, “periodontitis” and “intrabony defects”, which were adjusted to each database by necessity. Additionally, the bibliographies of the included studies and dissertations were searched for additional publications. The search language was restricted to English. After removing duplicates and completing the study selection process, titles and abstracts were scanned by 2 independent investigators according to predefined selection criteria, and potentially relevant RCTs were selected. Hard copies of all relevant articles were retrieved and read in full for further identification. Two reviewers independently extracted the data, and controversies were settled by consensus or discussion with a third author. The following variables were extracted from each included study: study design (author, publish year), sample size (patients and teeth sites treated), follow-up duration, and main outcomes. When inadequate information existed in the studies, it was essential to contact the first authors to obtain and clarify the relevant data, as specified by the standardized protocol.

### 1.3 Quality assessment

The methodological quality of the trials included was assessed by the Cochrane Collaboration tool. The following items were assessed: random sequence generation, allocation concealment, blinding of participants, personnel and outcome assessment, incomplete outcome measures, selective outcome reporting, and other bias. Two independent practitioners met and reviewed every entry for accuracy and consistency, and discrepancies were resolved by consensus.

### 1.4 Statistical analysis

The Cochrane Collaboration Review Manager software package (RevMan Version 5.2) was used to perform meta-analyses. The overall effect size of each intervention was calculated as the weighted average of the inverse variance for study-specific estimates.^[10]^ For dichotomous variables, odds ratios (ORs) with the corresponding 95% confidence interval (CI) were calculated, and the corresponding weighted mean difference (WMD) was used to estimate numerical variables. In the presence of considerable heterogeneity, the data were pooled using a random effect. Statistical heterogeneity among individual studies was evaluated based on Cochrane *Q* test and *I*^2^ index, and statistical heterogeneity was confirmed if *I*^2^ was above 75% and *P* < .10.^[11]^

## 2 Results

**Figure 1:**
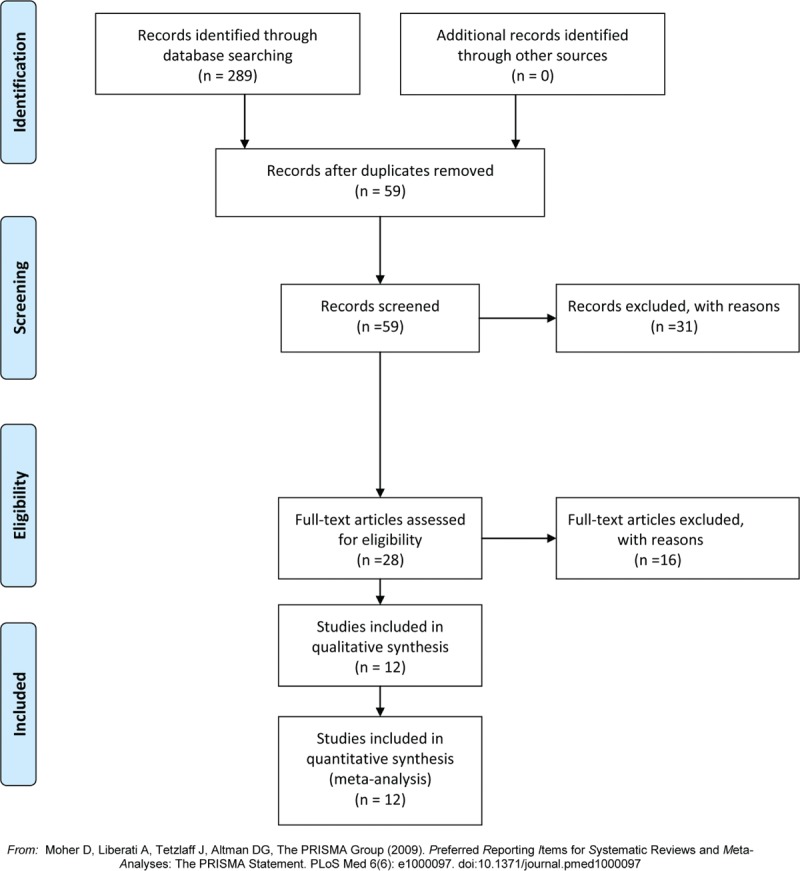
Flow diagram of the study selection.

Flow diagram contains a flowchart that describes the process by which we screened and selected trials. The initial literature search yielded 289 articles in total. The manual search of relevant reference did not identify any additional studies. Duplicate checking and title and abstract screening resulted in 28 publications. Finally, 12 intermediate- to high-quality studies were eligible for inclusion in the meta-analysis (Fig. 1).

**Figure 7:**
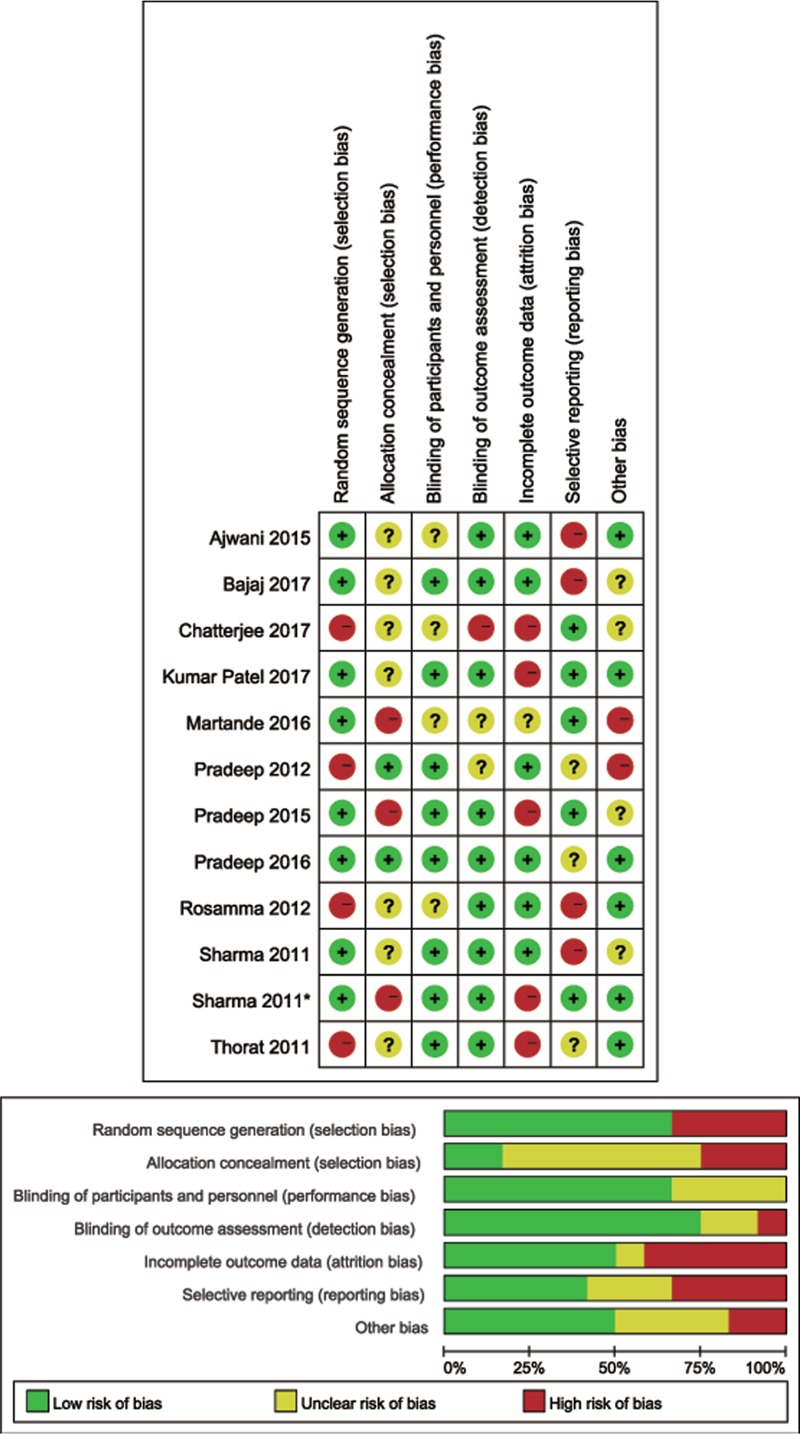
Risk of bias assessment of trials included.

Table 1 shows the detailed characteristics of the trials included. All studies reported the permission of the ethics and scientific committees of the participating centers. The detailed production method of PRF was reported in all studies. All treated cases in the studies showed uneventful wound healing. All patients in the included trials provided written informed consent before they underwent any study-related procedure. The appropriate sample capacity was calculated before the trials were conducted in all studies. The mean duration of follow-up ranged from 3 to 12 months. Detailed PRF preparation and surgical procedures were reported in all studies. Wound healing was uneventful for all treated cases in all studies included, and no adverse events occurred. Three study protocols were approved and registered under clinicaltrials.gov.^[6,7,12]^ Three studies were controlled clinical trials with a split-mouth design.^[2,8,13]^ All studies were single-centered and prospective trials. Based on the Cochrane Collaboration recommendation, randomization and comprehensive methodological processes were reported in 4 trials.^[2,5,12,14]^ Most studies reported detailed dropouts during interventions in consort flowcharts. To ensure adequate intraexaminer reproducibility, the examiner was calibrated before the beginning of the study in some trials.^[2,3,5–7,12,13,15]^ Three studies were placebo-controlled, triple-masked trials.^[2,12,14]^ Three studies were double–blinded clinical trials.^[13,15,16]^ The details of the risk of bias are illustrated in Figure 7.

**Table 1:**
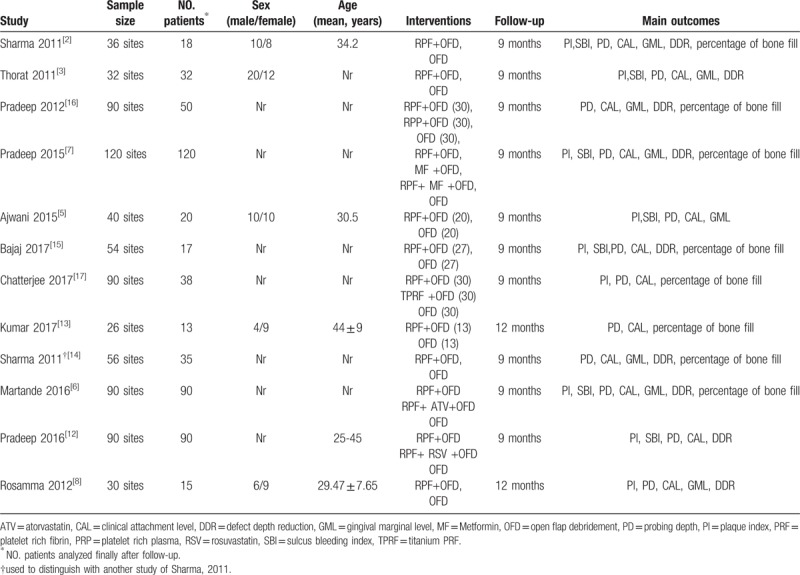
Summary of the characteristics of the trials in the included studies.

**Figure 2:**
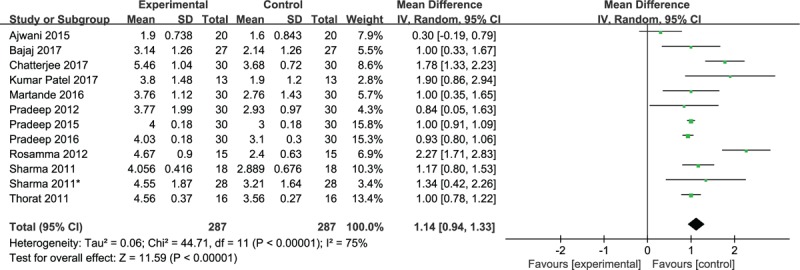
Forest plot for PD reduction. PD = probing depth.

Data regarding probing depth reduction for intrabony defects at 9 months postoperatively were reported in all 12 studies. There was no significant difference regarding probing depth (PD) reduction between the 2 groups in 1 study.^[5]^ Ten studies reported that significantly greater PD reduction was achieved in the PRF group compared with that in the OFD group. Similarly, pooled data found that adjunctive PRF precisely yielded significantly superior probing depth reduction for intrabony defects in chronic periodontitis patients postoperatively compared with OFD alone (WMD, 1.01; 95% CI 0.95– 1.08; *P* < .00001; heterogeneity, χ^2^ = 44.71, I2 = 75%, *P* < .00001) (Fig. 2).

**Figure 3:**
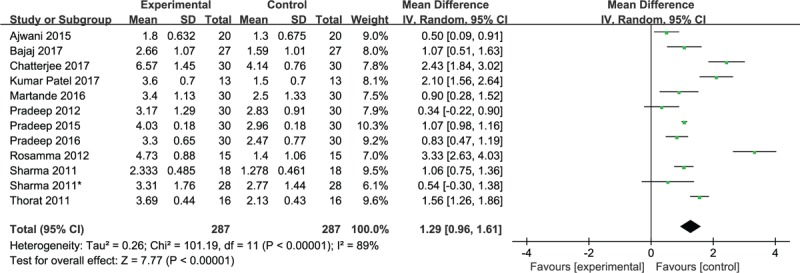
Forest plot for CAL gain. CAL = clinical attachment level.

Data regarding the gain in clinical attachment level (CAL) for intrabony defects postoperatively were available in 12 studies. Three studies revealed that no statistically significant difference was observed between the PRF and single OFD groups in terms of the gain in CAL for intrabony defects postoperatively (*P* < .05).^[5,14,16]^ Nine studies revealed that PRF plus OFD was superior to single OFD in terms of the gain in CAL after intervention. Even, the gain in attachment level in 1 study was significantly greater in the test sites, with a difference between the 2 groups in the relative vertical CAL (*P* < .001) and in the relative horizontal CAL (*P* = .001).^[2]^ Consistently, the pooled estimate found that the gain in CAL after treatment was higher in patients treated with PRF plus OFD than in OFD-treated patients (WMD, 1.29; 95% CI 0.–1.61; *P* < .00001). Considerable heterogeneity was found among the trial estimates (χ^2^ = 101.19, *P* < .00001), and the *I*^2^ index indicated that 89% of the variability across trials was due to heterogeneity rather than chance (Fig. 3).

**Figure 4:**
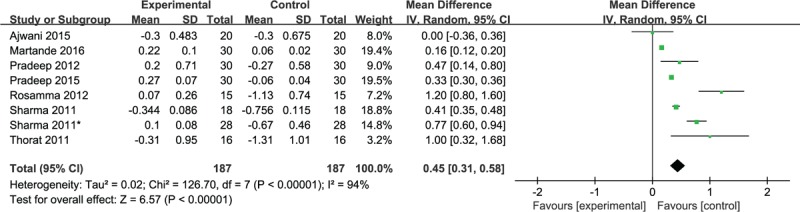
Forest plot for GML change. GML = gingival marginal level.

Eight studies reported the gingival marginal level (GML) with PRF and single OFD treatment after follow-up, of which 2 studies^[5,8]^ revealed no significant difference between the 2 groups postoperatively. The meta-analysis demonstrated that PRF was superior to single OFD with respect to GML change at the 9-month follow-up (WMD, 0.45; 95% CI 0.31–0.58; *P* < .00001), with considerable heterogeneity existing (χ^2^ = 126.7, *I*^2^ = 94%, *P* < .00001) (Fig. 4).

**Figure 5:**
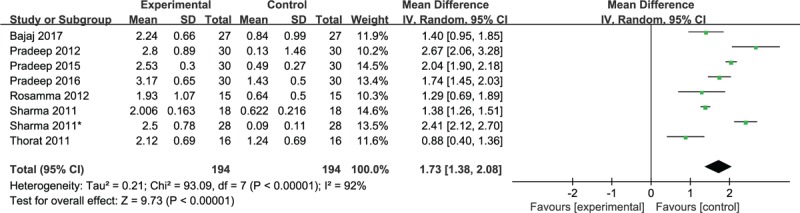
Forest plot for DDR. DDR = defect depth reduction.

Data regarding defect depth reduction (DDR) for intrabony defects after treatment for 9 months were reported in 8 studies. Notably, PRF plus OFD yielded statistically significant superior DDR after treatment compared with single OFD. Consistently, the pooled estimate revealed that more DDR was achieved in the PRF plus OFD group (WMD, 1.73; 95% CI 1.38–2.08; *P* < .00001). However, considerable heterogeneity was found (χ^2^ = 93.09, *I*^2^ = 92%, *P* < .00001). The random effects model was used for the meta-analysis (Fig. 5).

**Figure 6:**
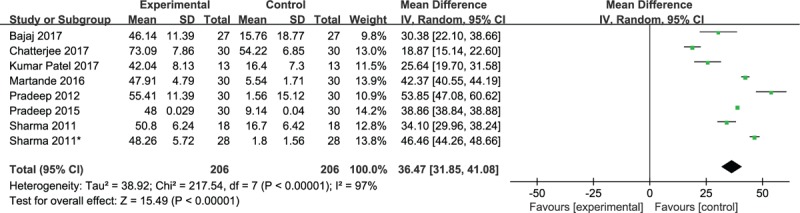
Forest plot for the percentage of bone defect fill.

Eight studies reported the percentage of fill defects in bone. Pooled data found that higher percentages of fill defects in bone were achieved in patients treated with PRF plus OFD compared with single OFD (WMD, 36.47; 95% CI 31.85–41.08; *P* < .00001; heterogeneity, χ^2^ = 217.54, *I*^2^ = 97%, *P* < .00001) (Fig. 6). When the control group (4.40 ± 1.04) and test group (4.52 ± 1.11) were compared, this effect was found to be nonsignificant at baseline (*P* > .05) and significant at the 9-month comparison (*P* < .05) in the study conducted by Thorat.^[3]^

Overall, plaque index was assessed in 8 studies. The means ± SD for plaque index were reported in 2 studies, and the difference between the test and control sites was statistically insignificant at the 9-month follow-up, although a significant reduction was observed in the other 2 studies.^[5,17]^ Thorat reported that the differences between 2 groups were statistically insignificant when compared at baseline and 9 months postoperatively (*P* > .05), as well as PI (plaque index) and reductions.^[3]^ PI was expressed as 3 levels, as follows: 0 to 0.5, 0.6 to 1.0, 1.1 to 1.5, in 6 studies.^[2,6,7,12,14,15]^ A statistically significant reduction in the PI and sulcus bleeding index (SBI) was observed in both test and control sites at 9 months postoperatively. However, an intergroup comparison suggested an insignificant difference in PI and SBI reduction. This finding indicates that an equivalent oral hygiene was maintained by patients of both groups throughout the study.

## 3 Discussion

To date, there have been 2 related systematic reviews^[18,19]^ on PRF plus OFD versus OFD for intrabony defects in chronic periodontitis patients, all of which clearly showed that adjunctive PRF was significantly beneficial to the administration of intrabony defects in terms of PD reduction, CAL gain, and bone fill. In addition, clinically significant improvements were achieved in periodontal parameters, such as CAL, intrabony defect, and probing depth reduction, when intrabony defects were treated with PRF monotherapy when compared to a traditional OFD procedure in another meta-analysis.^[20]^ However, fewer studies were included in the above meta-analyses. Several new clinical randomized controlled trials have been published in recent years. Therefore, an update meta-analysis was performed, and our findings are in agreement with those in previous articles.

Periodontal regeneration is a complicated process involving a number of different cell types and cell stromal interactions for complete regeneration.^[1,6]^ In the recent past, root-conditioning agents, bone replacement grafts, growth attachment factors and guided tissue regeneration procedures or combinations of these materials have been used for periodontal regeneration with various degrees of success.^[21]^ GML, CAL, PI, PD measurements and the presence of bleeding on probing are commonly used to assess and monitor periodontal status. To improve periodontal health, treatment aims to reduce PD, maintain or improve CAL, and reduce bone defect depth and the incidence of bleeding on probing. Probing depth reduction is the most desirable outcome variable of periodontal therapy. Gingival marginal position has a direct impact on the esthetics and long-term stability of periodontal therapy.

Platelet-rich concentrates are the most widely used regenerative biomaterials. In 1998, Marx et al first applied platelet concentrates in oral and maxillofacial surgery.^[22]^ Depending on the leukocyte and fibrin content, platelet concentrates were classified into 4 categories: pure platelet-rich plasma (P-PRP), pure platelet-rich fibrin (P-PRF), leucocyte- and platelet-rich plasma (L-PRP), and leukocyte and platelet-rich fibrin (L-PRF), such as Choukroun's PRF.^[5,23]^ The preparation of Choukroun's PRF is a simplified process, requiring no bovine thrombin, anticoagulants, or any other gelling agents.^[4]^ PRF acts as an immune regulation node with inflammation control abilities, including a slow continuous release of growth factors over a period of 7 to 14 days.^[24]^ Proteins derived from PRF included hepatocyte growth factor (HGF), transforming growth factor (TGF-b), platelet-derived growth factor (PDGF), vascular endothelial growth factor (VEGF), insulin-like growth factor (IGF), and epidermal growth factor (EGF).^[25]^

Autologous platelet concentrates, including PRP for surgical topical applications, are currently often used, but the quantification of the long-term growth factor release from these preparations in most cases is impossible. The overall benefit of PRF may be attributed to the dense fibrin matrix, which could transform a membrane approximately 1 mm in thickness and support growth factor release and cell migration.^[16]^ Its strong fibrin architecture and superior mechanical properties distinguish PRF from other kinds of platelet concentrates. Studies have proven a slower release of growth factors from PRF than PRP and observed better healing properties with PRF.^[26]^ A previous animal study showed that osteoblast cells treated with exudates of PRF collected at day 14 reached peak mineralization significantly more than the control group. The marked release of TGF-b1 and PDGF-AB from PRF resulted in the expression of alkaline phosphatase and induction of mineralization.^[27]^ Intrabony defects have also been found to exhibit pocket reduction and clinical attachment gain after 6 months with bone filling defects. In addition, the interaction between the fibrin matrix network and regenerative sites facilitates local endothelial cellular migration and is responsible for the vascularization, neo-angiogenesis, and survival of the adjunctive graft, with the abovementioned growth factor release. The high-interconnected fibrin network with a fine fiber structure in PRF originated from high thrombin concentration during its preparation.^[27,28]^

Wound healing was uneventful for all treated cases in all studies included, and no cases of flap dehiscence or infection were detected. A visual analog scale (VAS) was designed and used to assess the initial soft tissue healing in the study conducted by Rosamma et al.^[8]^ The higher score was associated with more severe inflammation (redness, edema, spontaneous bleeding, enlargement, or ulceration). Score 1 (33%) and score 2 (47%) were most often recorded in the PRF group, which was attributed to the high concentration of leukocytes. PRF could delay the blood activation process that could lead to increased leukocyte degranulation and the release of cytokines from proinflammatory mediators, such as tumor necrosis factor-alpha, interleukin-1 beta (IL-1β) and IL-6, to anti-inflammatory cytokines, such as IL-4.^[29]^ However, the main disadvantage of PRF is silica impregnation caused by the activation of the blood clot.^[4,8]^

The limitations of this systematic review involve restrictions on the publication language, the uniformity of the administration program and the small size of the included RCTs. Owing to few available data and confounding factors among studies, PI and SBI were not polled and just reviewed in this meta-analysis. In the present review, the retrieval language was limited to English, generating sampling bias. We used the Cochrane Collaboration tool to assess the risk of bias to evaluate the methodological quality of the included trials. A power analysis to determine the sample size prior to recruitment was performed in all studies. Intraexaminer calibration was performed to avoid examiner-related bias in all studies. Moreover, the follow-up ranged from 9 to 12 months and varied slightly in the studies included for the meta-analysis. Consequently, most studies were intermediate to high quality. However, trials with a high or unclear risk of bias could lower the quality of evidence in our results.^[30]^ The results of the systematic analysis did not demonstrate any significant heterogeneity among different studies. In addition, owing to small, single-center trials per treatment group, a center effect was inevitable, which could be excluded by comparing the cases enrolled in 2 or more participating institutions. The high-level evidence needs wide strictness and consistency to support.

## 4 Conclusions

Adjunctive use of PRF with OFD significantly improves fill defects when compared to OFD alone. However, additional powered studies with much larger sample sizes are needed to obtain a more concrete conclusion. Although the interpretation of the study results was limited, we believe that to a certain extent, our analyses may provide valuable information for physicians who need to decide the best treatment strategy among all possible regimens for patients with intrabony defects.

## Author contributions

**Data curation:** Ang Li, Hongjie Yang.

**Formal analysis:** Hongjie Yang, Hongqiang Wang.

**Investigation:** Jingyi Zhang.

**Methodology:** Jingyi Zhang, Shulian Chen.

**Project administration:** Shulian Chen.

**Resources:** Yanzheng Gao, Hongqiang Wang.

**Software:** Hongqiang Wang.

**Validation:** Ang Li.

**Writing – original draft:** Ang Li.

**Writing – review & editing:** Yanzheng Gao.
